# Primary cutaneous melanoma of the breast: A case report

**DOI:** 10.1186/1757-1626-1-212

**Published:** 2008-10-04

**Authors:** Ahmed Alzaraa, Narinder Sharma

**Affiliations:** 1 Department of General surgery, Calderdale Royal Hospital, Halifax, UK

## Abstract

**Background:**

Primary cutaneous melanoma of the breast is a very rare tumour, accounting for < 5% of all malignant melanomas.

**Case presentation:**

A young lady was seen in the breast clinic for a skin lesion in the right breast. Clinical examination and investigations confirmed a diagnosis of a primary cutaneous melanoma of the breast. The lesion was excised and the patient made good recovery. She has shown no signs of local recurrence and is under regular follow-up in the dermatology clinic.

**Conclusion:**

This case is educational as it shows that the treatment of breast cutaneous melanoma is similar to that for any skin parts with surgery remaining the main therapeutic option. It also shows that mastectomy is unnecessary as it does not improve the results obtained by wide local excision of melanoma.

## Background

Primary cutaneous melanoma rarely affects the breast, accounting for less than 5% of all malignant melanomas. Operable cutaneous melanomas are treated with wide local excision. The presence or absence of metastases in the regional lymph nodes (RLN) is the most significant prognostic variable that predicts survival in patients with melanomas.

## Case presentation

A 22 years-old female was assessed in the breast clinic in November 2004 for a right breast skin lesion which had been present for about a year. It had increased in size and occasionally bled. She is non smoker and has no significant past breast history. There is no family history of breast cancer.

**Figure 1:**
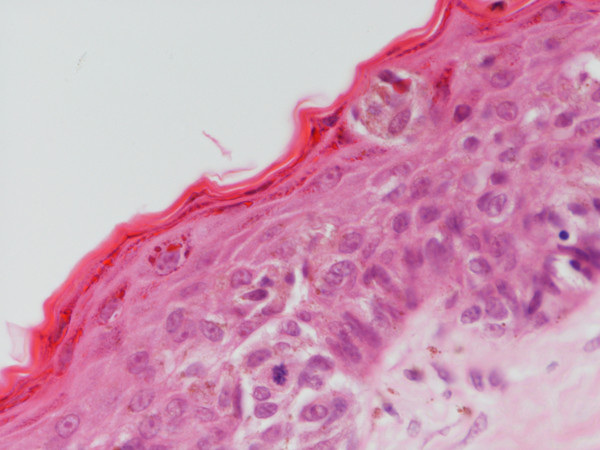
Scattered atypical melanocytes showing mitotic activity in the epidermis (HE,400×).

Examination revealed a 5 mm mole on the right areola without any associated breast lumps or palpable axillary lymph glands. No organomeagaly was noted on abdominal examination. Excision of the mole under a local anaesthetic in January 2005, showed it to be a superficial spreading malignant melanoma with a tumour thickness of 0.5 mm, corresponding to pT1, Clarke level II (Figure 1). Minimum lateral clearance was 1 mm with a 4 mm deep clearance. CT of abdomen and chest excluded the presence of a metastatic disease. She underwent wider excision but no residual disease was found, and it also resulted in a clearance margin of more than 1 cm. Twelve months post surgery, she remains well in herself under the care of the dermatologists without any signs of recurrent/metastatic disease.

## Discussion

Primary cutaneous melanoma rarely affects the breast, accounting for less than 5% of all malignant melanomas [1,2]. Several cases have been reported in the literature [2,3]. It is highly probable that there is no significant difference between melanomas of the breast and melanomas arising from other cutaneous areas as far as correlation of Clark's level of invasion with prognosis and regional lymph node status is concerned [1,3]. In general, operable cutaneous melanomas are treated with wide local excision. 1 cm clearance is adequate for lesions < 1 mm thick and a 2 cm margin for lesions up to 4 mm thick [4]. There are few data to support the use of margins wider than 2 cms even in lesions > 4 mm thick [5]. In our case the radial margin of over 1.0 cm was adequate as the lesion was < 1 mm thick. The presence or absence of metastases in the regional lymph nodes (RLN) is the most significant prognostic variable that predicts survival in patients with melanomas. It is extremely rare for it to spread systemically without first passing through the first draining lymph node basin [6]. These neoplasia are felt to follow a different metastatic pattern than do primary carcinoma of the breast, and require a different therapeutic approach [3].

Papachristuo et al [3] found that lesions located below a 3 cm radius from the clavicle metastasised exclusively to the axillary lymph nodes regardless of sex or location. Further, in their 19 patients with central and medial lesions where internal mammary nodes were available for examination, none of them had disease in those nodes despite the fact that half of them had simultaneous axillary node metastases. Where the RLN are impalpable, their surgical assessment has been controversial and has varied from selective lymph node dissection to elective lymph node dissection (ELND). A major argument against ELND in all is that if all patients with high risk melanomas are subjected to ELND, 70–80% will receive an unnecessary surgical procedure as only 20–30% will have RLN metastases. The therapeutic benefit of removing clinically normal nodes has never been proven [7,8]. Patients with thick melanomas (> 4.0 mm) have a high incidence of systemic disease and should undergo a proper extent of disease evaluation. Regional node assessment provides valuable staging and prognostic information [4], and for lesions located 3 cm from the clavicle [3], nodal assessment on the cervical region has been recommended. Assessment of regional nodes was not necessary in our case as the tumour was < 1 mm thick. Just over a decade ago regional nodes were assessed by complete lymph node dissection (CLND) [6] resulting in unnecessary surgery in many [9,10]. However, the introduction of sentinel lymph node biopsy(SNLB) into surgical practice has revolutionized the assessment of regional nodes [10]. Morton and colleagues were the first to demonstrate that lymphatic drainage from a melanoma can be "mapped" by injecting the skin around the tumour with blue dye[10]. Injected blue dye was shown to travel through lymphatic channels to the first, or "sentinel" lymph node (SLN) that drains the tumour. It was shown that histological examination of the SLN accurately reflected the pathological status of the entire regional lymph node basin. A positive SLN is associated with a higher chance of the remaining nodes containing metastases, while patients with negative SLNs infrequently have other nodes that contain tumour cells [9,10]. SLN mapping, therefore, spares approximately 80–85% of patients with melanoma from having CLND for what will ultimately be a negative regional nodal basin [9,10].

Patients with melanomas most likely to benefit from SLNB have thickness of 1–4 mm [10] as the incidence of nodal metastases ranges from 6% for patients with 1 mm melanomas to 35% in patients with 4 mm melanomas [10,11]. For patients with melanomas less than 1 mm, the indications for SLNB would be Clark level IV depth, presence of tumour regression, and ulceration [12]. Young age, higher number of mitoses, male gender and axial location are viewed as some of the relative indications [13]. The third interim analysis of the Multicentre Selective Lymphadenectomy Trial (MSLT-1) on SLNB shows that melanoma patients who had wide excision followed by selective lymph node dissection had a survival similar to patients where a watch and wait policy had been followed, with complete lymph node dissection in case of pathological lymph node involvement. In this study the disease free survival was superior after SLNB [14].

## Conclusion

Treatment of breast cutaneous melanoma is the same as that for any skin parts. Surgery remains the most important therapeutic modality particularly in preventing local recurrence. Mastectomy does not improve the results obtained by wide excision of the primary melanoma. SLNB, with tremendous benefits and minimal risk, is becoming widely adopted as most surgeons who treat breast cancer and melanoma incorporate this technique into their practice.

## Competing interests

The authors declare that they have no competing interests.

## Authors' contributions

AA: Searched literature, revised and edited manuscript. NS: Edited manuscript. All authors have read and approved the manuscript.

## Consent

Informed written consent was received for publication of the manuscript and figures.
